# An Unusual Rash in a Five-Year-Old Girl: Blaschkoid Distribution Is the Key to the Diagnosis

**DOI:** 10.7759/cureus.12124

**Published:** 2020-12-17

**Authors:** Jayasree Vasudevan Nair, Giridhar Guntreddi, Swayam Nirujogi

**Affiliations:** 1 Pediatrics, Sutter Health, Jackson, USA; 2 Pediatrics, Sanford Health, Bemidji, USA; 3 Family Medicine, Tower Health Medical Group, Reading, USA

**Keywords:** lichen striatus, blaschkoid lines

## Abstract

Lichen striatus is a rare dermatological condition seen in children. The exact etiology of this self-limiting eruption is unknown. A combination of genetic predisposition with an infectious trigger is the most accepted hypothesis for the etiology. Treatment is typically not necessary, as the disease is self-limiting. Treatment options with topical low to mid-potency corticosteroids may be used for symptomatic treatment of pruritus, however, it does not alter the course of the disease or post-inflammatory dyspigmentation. Successful treatment of skin lesions with calcineurin inhibitors is reported in isolated studies. The rapidly growing lesion of lichen striatus can cause considerable parental anxiety. Familiarity with this condition for primary care pediatricians is necessary to make the correct diagnosis and to alleviate parental anxiety. Here, we present a case of lichen striatus albus, a variant of lichen striatus, in a five-year-old girl presenting as a skin rash.

## Introduction

Dermatological conditions are a common reason for pediatric office visits. The significance of the lesions can vary from a completely benign condition to a marker of serious underlying systemic diseases. In this article, we present a case of Lichen striatus albus, an uncommon, benign dermatological condition, in a five-year-old girl. Lichen striatus is an inflammatory dermatosis distributed along the lines of Blaschko, most commonly affecting children [1]. Diagnosis is usually clinical based on the presentation and morphology of the skin lesions. The typical appearance is flat-topped lichenoid, red, skin-colored, or hypopigmented papules coalescing into a linear band. The time taken for the band to reach its maximum length is variable from a few weeks to several months. The large skin lesion of lichen striatus enlarging for months can cause parental concern and anxiety. Correct diagnosis of the condition and reassurance can alleviate parental anxiety and avoid unnecessary referrals, resulting in better patient care.

## Case presentation

A healthy five-year-old girl presented to the pediatric clinic along with her anxious mother to evaluate a rash. About two months ago, the mother noticed a few whitish papules on her daughter’s right upper thigh. The mother denied any preceding illness, allergy, or any possible triggers. Since then, the rash grew along the entire length of her right thigh in a linear fashion. She denied pain or pruritus or hair loss. There was no rash anywhere else on her body and the nails were not affected. The girl has been otherwise well. Her past history is negative for allergy, asthma, eczema, and otherwise unremarkable, as was her family history. She was not on any regular medications. She did not receive any topical or systemic treatment for the rash. She was afebrile and had normal growth parameters. Other than the rash, which is described, her vital signs and physical examination were within normal limits.

**Figure 1:**
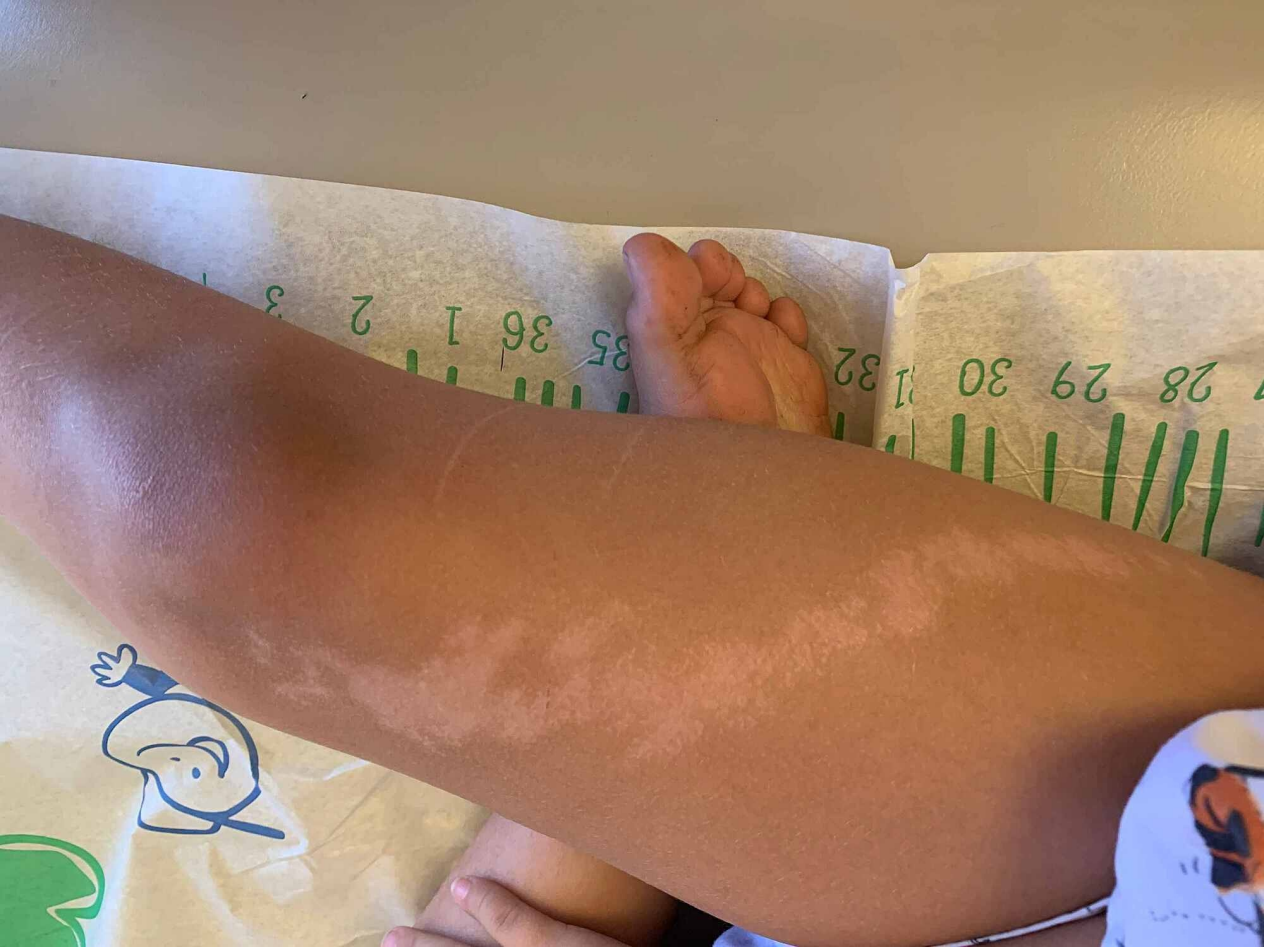
Rash of lichen striatus albus on the right lower extremity

The lesion was a 14 cm long linear band consisting of macules and 1-2 mm-sized flat-topped papules(see Figure 1). The band seems to be following the lines of Blaschko. The lesion extended from her upper right thigh to below the right knee. The lesion was hypopigmented, minimally palpable, non-scaly, non-tender, and non-pruritic. No rash was noticed on other parts of her body. Her nails appeared normal. Based on the appearance and clinical findings, the lesion was diagnosed as lichen striatus albus. Mother was reassured about the benign nature of the condition and explained that the rash may take several months or even years to resolve. Available treatment options, including corticosteroid creams, and their inability to alter the course of illness was discussed with the mother. The role of newer calcineurin inhibitors was also discussed. The mother opted not to treat her child at this point.

## Discussion

Lichen striatus is a relatively rare condition, first described by Senear and Caro in 1941 [1]. It typically affects children between the ages of five and 15 years, and more than 50% of the affected are children [1]. It may occasionally affect infants and adults, and no age is exempt. Some studies reported a higher incidence in preschool children and during summer months [1]. Female sex predilection is reported in some case series [1]. No racial variation is noted [2], and the disease distribution is worldwide with cases reported from several countries.

Etiology and pathogenesis

The etiology and pathogenesis of lichen striatus are mostly unknown. Many triggers like viral infections, hypersensitivity reactions, vaccinations, trauma, medications, and pregnancy are implicated as predisposing factors [2]. Concurrent occurrence with vitiligo and pityriasis rosea are reported [2]. Adalimumab and etanercept are implicated as triggers [2]. Many authors observed a high incidence in people with a personal or family history of atopy [2]. Familial cases are also reported in the literature [3].

Many hypotheses are suggested for the pathogenesis of the condition. Because they follow the lines of Blaschko, a theory of post-zygotic somatic mutation in keratinocytes during early embryogenesis is suggested [4]. These clones may remain dormant until a predisposing event upsets the immunologic tolerance and initiates an autoimmune response. The finding of cluster of differentiation 8 (CD8) T lymphocytes surrounding the necrotic keratinocyte and activated Langerhans cells may suggest a cell-mediated immunity in its pathogenesis [4].

Blaschko lines are an interesting concept first described by German dermatologist Alfred Blaschko in 1901 [5]. They are clinically important, as many inherited and acquired diseases follow these lines, making a characteristic visual appearance. These invisible lines are believed to have an embryologic origin, and they correspond to epidermal cell migration pathways. The morphology of these lines are linear on extremities, V-shaped over the spine, and S-shaped on the lateral and anterior aspect of the trunk, and they don’t cross the midline. They are not related to anatomic landmarks based on nerves, blood vessels, or lymphatic distribution.

Clinical features

Lichen striatus usually presents as a sudden eruption of pink, red, skin-colored, or hypopigmented flat-topped papules arranged in a linear continuous or interrupted band [6]. The lesion may be slightly scaly. It may be asymptomatic or pruritic. Occasionally, vesicular elements may be noted. The band may be several centimeters in length. The lesion continues to grow for a variable time - a few weeks in many cases and, occasionally, it may continue to grow for several months. Lichen striatus lesion follows the lines of Blaschko. The lesion is usually unilateral, but bilateral lesions are reported [6]. The extremities are the commonly affected site followed by the trunk, buttocks, face, and nails, in that order.

There are three morphological variants of lichen striatus: typical lichen striatus, lichen striatus albus, and nail lichen striatus [6]. Approximately 80% of patients present with the typical variant [6]. The lichen striatus albus variant consists of hypopigmented macules and/or papules and is more common in dark-skinned patients. A study conducted on 115 patients observed that lichen striatus albus was rare and constituted 15.6% percent of the cases [7]. Other than the morphology, clinical course and other characteristics are identical for typical lichen striatus and lichen striatus albus. Nail lichen striatus affects the nail matrix, presenting as onycholysis, leukonychia, nail pitting, splitting, ridging, fissuring, and nail plate thinning or thickening. Lichen striatus typically affects only one nail involving the lateral and medial portion of the nail plate. It can present along with skin lichen striatus or as an isolated nail condition.

The clinical course of lichen striatus is variable. The active phase lasts about three months to several years. Spontaneous resolution is the rule, leaving post-inflammatory hypopigmentation or rarely hyperpigmentation. Hypopigmentation is reported in 25%-59% of lichen striatus patients [7]. The dyspigmentation would resolve in one to three years, which is not typically affected by treatment. Nail lesions resolve after several years without permanent nail dystrophy. Relapses are rare, but if it happens, it affects the same side of the body.

Lichen striatus can be diagnosed based on clinical features. In doubtful cases, a skin biopsy may be helpful. Histopathological features depend on the age and anatomic location of the lesion [7]. Even though the findings can be nonspecific, distinct features are present during the active phases, supporting the diagnosis. The dermal-epidermal junction of the papillary dermis shows superficial, band-like lichenoid infiltrates with focal vacuolar alteration of the basal layer [7]. Dermal lymphocytic infiltrates surrounding the hair follicles and eccrine glands are also characteristic [7]. Focal spongiosis, exocytosis, necrotic keratinocytes, patchy hyperkeratosis, and dyskeratosis are changes noted in the epidermis [8].

Many dermatological conditions mimic lichen striatus (Table 1).

**Table 1:** Differential diagnosis

Inflammatory linear verrucous epidermal nevus
Linear morphea
Linear lichen planus
Linear cutaneous lupus erythematosus
Linear porokeratosis
Linear Darier disease
Lichen nitidus
Linear graft versus host disease
Blaschkitis

Linear lichen planus (LLP) is a rare variant of cutaneous lichen planus preferentially affecting children. The pruritic purple polygonal papules of LLP are arranged in a linear fashion along the lines of Blaschko, making it a top differential diagnosis for lichen striatus. However, in contrast to lichen striatus, the LLP lesions are typically violaceous, larger, and can be hypertrophic [9]. The nail lichen planus affects multiple nails, unlike the nail lichen striatus, which affects only one nail. Diagnosis is usually clinical, but a skin biopsy is helpful in doubtful cases, as histopathology can differentiate the two conditions. Histopathology shows cytoid bodies at the dermo-epidermal junction and positive direct immunofluorescence to multiple immunoglobulins. Typically, the skin condition would go into remission in one to two years. Treatment is considered for symptomatic relief.

Inflammatory linear verrucous epidermal nevus (ILVEN) is a variant of the epidermal nevus. It is characterized by unilateral erythematous, pruritic, inflamed, and hyperkeratotic plaques that grow in a linear fashion. It commonly affects the lower extremity. It can present at birth but usually presents in early childhood. ILVEN is a challenging differential for lichen striatus. Contrary to lichen striatus, ILVEN consistently presents with pruritic rash [9]. It doesn’t regress spontaneously; its course is characterized by periods of remission and exacerbations.

Linear morphea can cause diagnostic difficulties with lichen striatus, especially during the early stages. It affects children in the age group of five to eight years. But can affect younger age groups and even present as a congenital skin condition. It affects the face and extremity of children. Firmness and induration of the lesion may help differentiate it from lichen striatus. Even though the early disease's histopathology may have overlapping features with lichen striatus, linear morphea in the late stages shows epidermal atrophy and dermal sclerosis. There are case reports of facial lichen striatus preceding linear morphea warranting the facial lichen striatus to be followed until complete resolution [10]. Linear morphea doesn’t resolve spontaneously. It can lead to scarring, disfigurement, and atrophy of affected extremities. Associated extracutaneous manifestations may be present in a subset of children.

Hypomelanois of Ito is another differential, particularly for lichen striatus albus. This condition is believed to be caused by a mutation during early embryogenesis. It may be present at birth or noticed during the first few years of life. The characteristic skin lesion shows small hypopigmented white macules at presentation. Eventually, they become confluent, forming large streaks and swirls of hypopigmentation, which follow the lines of Blaschko. Associated extracutaneous manifestations of the disease commonly affect the central nervous system (CNS) and musculoskeletal system [11]. The absence of a papular element, more extensive affectation, and extracutaneous manifestations differentiate it from lichen striatus. In doubtful cases, a skin biopsy may be helpful.

Management

As lichen striatus is a self-limited condition and usually asymptomatic, treatment is not typically necessary. Topical corticosteroids can be used for symptomatic relief of pruritus [12]. But this would not affect the duration of the eruption or dyspigmentation. Successful treatment with calcineurin inhibitors are reported [13]. Excimer laser for the treatment of hypopigmentation is used in a small number of cases [14]. The recovery in nail lichen striatus may be hastened by intralesional triamcinolone injection [15]. Correctly diagnosing the condition and reassuring the parents are the crucial factors in the management. The facial lichen striatus is to be followed up closely, as there are reports of lichen striatus preceding linear morphea in children.

## Conclusions

In this case report, we present a case of lichen striatus albus, a rare morphological variant of lichen striatus in a five-year-old girl. Lichen striatus albus shares the same clinical characteristics as the other forms of lichen striatus other than the hypopigmentation of the eruption. In our case, distribution along the lines of Blaschko and characteristic morphology helped us reach a clinical diagnosis of lichen striatus albus. However, in situations of uncertainty, a biopsy would be helpful. The mother of the patient was reassured about the self-limiting nature of the rash, and she opted for no treatment. Many dermatological conditions in children, such as inflammatory epidermal nevus, linear lichen planus, linear morphea, linear cutaneous lupus, and hypomelanosis of Ito, show a Blaskhoid distribution. An understanding of the Blaschko lines and Blaschkoid dermatosis would help clinicians to narrow down the differentials. This case report would help pediatricians familiarize themselves with Blaschko line dermatosis and lichen striatus to make the correct diagnosis in such cases. An approach with sound clinical judgment would help avoid unnecessary treatment and parental anxiety and reduce healthcare costs in primary care settings.
